# Data collected to assess the effect of inquiry-based learning on environmental knowledge and attitudes among pre-service biology teachers in Tanzania

**DOI:** 10.1016/j.dib.2023.109429

**Published:** 2023-07-18

**Authors:** Josephat Paul Nkaizirwa, Catherine Musalagani Aurah, Florien Nsanganwimana

**Affiliations:** a African Centre of Excellence for Innovative Teaching and Learning Mathematics and Science (ACEITLMS), University of Rwanda, College of Education (URCE), Kayonza, P.O. Box 55, Rwamagana, Rwanda; b Department of Educational Psychology and Curriculum Studies, College of Education, The University of Dodoma, P.O. Box 523, Dodoma, Tanzania; c Department of Science and Mathematics Education, School of Education, Masinde Muliro University of Science and Technology, Kakamega, P.O. Box 190-50100, Kenya

**Keywords:** Competence Model for Environmental Education, Two-Major Environmental Values (2-MEV), Teacher colleges, Solomon four-group design, Environmental literacy

## Abstract

The proliferation of global environmental problems has necessitated the need to re-examine the environmental impacts caused by human-nature relations. Yet individual attitudes and environmental literacy remain among the critical determinants of environmental sustainability. Consequently, environmental psychology has been one of the most effective tools for shaping people's actions in favor of the environment. As such, this article presents a dataset that describes an intervention carried out to explore the effect of inquiry-based learning on shaping environmental attitudes (EAs) and knowledge of pre-service biology teachers in selected teacher colleges (TCs) in Tanzania. Data were collected from four TCs located in four different regions of Tanzania (N = 333). Particularly, EAs were measured using the two-factor model of ecological attitudes (2-MEV) by assessing two uncorrelated domains of environmental attitudes: Preservation and Utilization of nature. In addition, a Competence Model for Environmental Education was used to measure the three dimensions (system knowledge, action-related knowledge, and effectiveness knowledge) of environmental knowledge among pre-service biology teachers. A modified design of the Solomon four-group was employed to guide the intervention in measuring the level of change in EAs and environmental knowledge before and after the study intervention. A paired-sample *t*-test was used to assess the effect of the intervention on TCs that had pre- and post-tests, given the variation in the number of subjects in each TC. In addition, the one-way analysis of variance (ANOVA) compared the mean scores of the combined study groups at the post-test. Besides, in the regression analysis, Hayes' PROCESS macro (version 4.1) was used to assess the moderation effect of the Social Desirability Responding (SDR) on the relationships between EAs and environmental knowledge. Likewise, age (in years) was entered as a covariate in the regression model of the Statistical Product and Service Solution (IBM SPSS version 25). The presented dataset can act as a basis for improving the status of environmental education delivery in teacher education in Tanzania and other areas with similar or related contexts. Thus, program-specific interventions could be designed for prospective biology teachers as potential change agents in shaping how people interact with the environment.

**Specifications Table**

**Table:** 

Subject	Sustainability education
Specific subject area	Environmental attitudes and environmental knowledge
Type of data	Table Graph Figure
How the data were acquired	Data were acquired through the Environmental Knowledge Test, the 2-MEV model, and the Balanced Inventory of Desirable Responding Short Form (BIDR-16). Differentiated data collection methods were used. Particularly, pre- and post-intervention measurements were used to obtain data from 160 pre-service biology teachers, whereas the post-intervention measurement was employed to collect data from the remaining 173 participants in two other TCs whose participants had post-tests only. All the participants were obtained using single-stage cluster sampling from four out of ten TCs offering science-related diploma programs.
Data format	Raw Analyzed
Description of data collection	Data were collected from January to April 2021 using questionnaires and an environmental knowledge test. Before the data collection, the sampled TCs were randomly assigned to treatment (for intervention) and comparison groups (conventional approach). Pre- and post-intervention measurements were administered to the selected groups, while other subjects received post-intervention measurements only. The intervention lasted for three weeks in each of the intervention TCs.
Data source location	The TCs from which data were collected were located in four different regions (Mwanza, Iringa, Tanga, and Dodoma) of Tanzania. However, a pilot test of the research tools was conducted in a separate TC in Morogoro, Tanzania.
Data accessibility	The dataset titled “Data for assessing EA and knowledge in selected teacher colleges in Tanzania” is publicly available at:
	Repository name: Mendeley Data Repository Data identification number: DOI: 10.17632/5234245rv.1 Direct URL to data: https://data.mendeley.com/datasets/52342x45rv/1 And “Tools for data collection and scores of pre-service biology teachers” available at: https://data.mendeley.com/datasets/ww6vn5v3dy/1 Data identification number: DOI: 10.17632/ww6vn5v3dy.1
Related research article	J. P. Nkaizirwa, C.M. Aurah, F. Nsanganwimana, An empirical investigation of environmental knowledge and attitudes as the correlates of environmental identity among pre-service biology teachers in Tanzania, Sustainability. 15(1) 669. 10.3390/su15010669.

## Value of the Data

• The current data provide fundamental insights on the effectiveness of differentiated facilitation strategies in enhancing environmental attitudes and knowledge. As such, the presented data call for the need to rethink the facilitation strategies that engage learners outside the four walls of the classroom setting, particularly for exploring environmental aspects that are context-specific.
• Practitioners of environmental psychology can benefit from the presented data, particularly in the design of the program-specific intervention for enhancing environmental stewardship in Tanzania and other related contexts. This speaks to the unique orientation displayed by the Utilization and effectiveness domains in the presented data, thereby attracting new debates for future research.
• The validated items of the 2-MEV model and the test questions in a Competence Model for Environmental Education can serve the fundamental purpose of psychometric properties in measuring environmental attitudes and knowledge of people from non-western countries, predominantly Sub-Saharan Africa and Tanzania in particular.

## 1 Objective

The primary purpose of generating this dataset is to document the impact of exposing pre-service biology teachers to different exploratory opportunities in their preparatory stages as prospective environmental educators. Specifically, using differentiated instructional strategies in understanding the higher-order factors of environmental attitudes (Preservation and Utilization) and their relationship with other environmental variables, particularly the general cognitive structure of environmental knowledge dimensions. Additionally, the generated dataset aims, among others, to document the need to control for SDR as a potential moderating factor when measuring self-reported environmental constructs such as environmental attitudes.

## 2 Data Description

This dataset is composed of descriptive and inferential statistics on the effect of using differentiated instructional methods on enhancing the EAs and environmental knowledge of pre-service biology teachers in TCs, Tanzania. The research from which the dataset was generated was designed to provide information on the impact of using inquiry-based learning and conventional methods to enhance EAs and environmental knowledge among pre-service biology teachers from different TCs. This dataset is divided into four major sections, as described underneath, based on the variables that generated it.

### 2.1 Formulation and Contextualization of the 2-MEV Items

For measuring EAs, 20 items were adapted from the 2-MEV model, out of which 10 items measured the Preservation factor (e.g., *We must set aside areas to protect endangered species*), whereas the other 10 items measured the Utilization factor [1]. Preservation is a biocentric-oriented factor that measures the extent to which people endorse items related to the protection and conservation of nature (e.g., *nature is always able to restore itself*). On the other hand, the Utilization factor measures the anthropocentric perspective, whereby people prioritize the use of environmental resources for their own benefit and consider human beings superior to other creatures within the environment. Before the actual data collection process, the items were pilot-tested and modified to suit the Tanzanian context.

Given a thorough data cleaning after the actual data collection and after testing for psychometric properties, only 13 items were validated and used to generate the current dataset. Again, the structure of the generated dataset and specific wording is presented in Mendeley Data Repository files named “Tools for data collection and scores of pre-service biology teachers” (available through the following link: https://data.mendeley.com/datasets/ww6vn5v3dy/1). This file contains a dataset of EA and its related scores from the participants, as well as other data for environmental knowledge and the social desirability responding (SDR) scale, as explained next. Participants rated each item on a five-point scale from 1-strongly disagree to 5-strongly agree. The scores were aggregated to obtain the cumulative score of each factor, which was then compared with other variables for inferences.

### 2.2 Formulation and Contextualization of the EKT Items

The Environmental Knowledge Test (EKT) items were developed, modified, and used to measure three dimensions of environmental knowledge [2]. Particularly, the three dimensions measured included; system knowledge (knowledge about the natural operation of environmental systems), action-related knowledge (best ways to achieve resource conservation and nature preservation), and effectiveness knowledge (knowledge about the effectiveness of various actions to achieve nature conservation) [3]. Consequently, the presented dataset was obtained after a thorough review, a pilot test, and after validating the employed items for future use. The items comprised multiple items and other open-ended questions that required participants to respond without explaining them or explaining the cause and effect of environmental events. The EKT is available in the Mendeley Data repository through the same link with the same file name mentioned in the previous section (at: https://data.mendeley.com/datasets/ww6vn5v3dy/1). The decision on retaining or modifying the items relied on item analysis as recommended by Quaigrain and McCowan [4,5].

### 2.3 Formulation and Contextualization of the BIDR Items

The Balanced Inventory of Desirable Responding short form version was adapted from Hart et al. [6] and it was used to control the effect of social desirability responding in the presented dataset. The BIDR-16 was developed as a revised version of the 40 items that were initially developed but revised after being considered difficult to complete and time-consuming [7]. The current version is easy to complete and is effective in controlling SDR on self-rated scales. As in the previous section, the BIDR items were adapted and modified to suit the Tanzanian context. Particularly, one item (i.e., *doubted ability as a lover*) was removed from the scale for contextual irrelevance. Participants rated the items on a five-point scale from 1-strongly disagree to 5-strongly agree.

### 2.4 Moderation Effect of SDR on the Relationships Between Environmental Knowledge and Environmental Attitudes

**Fig. 1:**
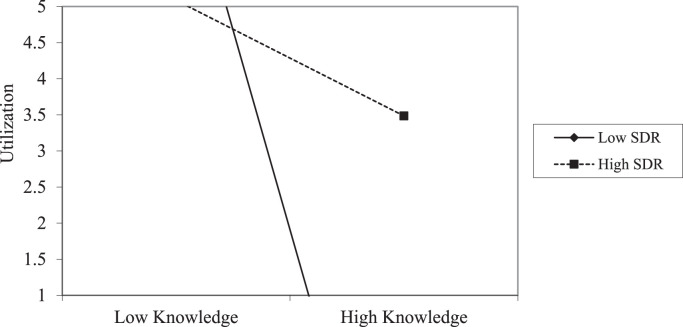
Indicates the interaction effect of SDR on the relationship between environmental knowledge and utilization in TC_1_ at the pre-test.

**Fig. 2:**
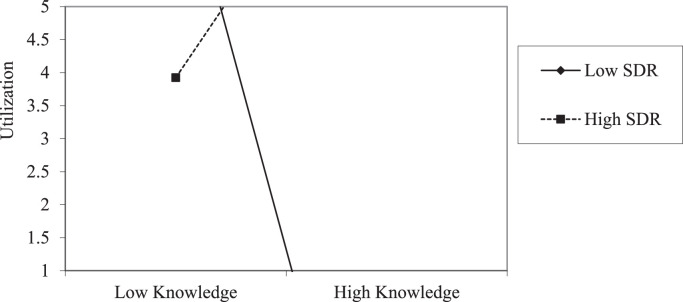
Indicates the interaction effect of SDR on the relationship between environmental knowledge and Utilization in TC_2_ at the pre-test.

Apart from measuring the usability of the tools employed in the presented data, an effort was made to assess the moderation effect on the relationships between environmental attitudes (EAs) and environmental knowledge. Specifically, the moderation effect on the relationships between EAs and environmental knowledge was assessed by using the Hayes’ PROCESS macro (version 4.1) embedded in the Statistical Product and Service Solution (SPSS version 25). Besides, the age (in years) of the participants was entered in the model as a covariate. Before variables were entered into the regression model, all independent variables were mean-centered, followed by the creation of a product term for measuring the interaction effect on the dependent variable [8]. Additionally, the data were subjected to normality tests, homoscedasticity, and test of independence residuals before conducting the inferential statistics. Accordingly, Tables 1 and 2 provide summaries of the resulting dataset when the relationships between environmental knowledge and EAs were assessed before the intervention process in teacher colleges one and two (TC_1_ and TC_2_). Besides, Figs. 1 and 2 provide additional information depicting the level of the interaction effect of SDR on the relationships between EAs and environmental knowledge dimensions in TC_1_ and TC_2_ before the intervention. The analysis was conducted twice, at the initial and at the final stages of the data collection. The resulting dataset was compared before and after the intervention based on the study groups, as explained in the subsequent sections.

**Table 1:** Indicates the moderation of SDR on the relationship between environmental knowledge and environmental attitudes in TC_1_ at the pre-test.

Variables	Coeff.	se	t	p	LLCI	ULCI
IV: Preservation						
Constant	0.4524	1.4174	0.3192	0.7504	-2.3653	3.2701
Knowledge	0.3369	0.3455	0.9750	0.3323	-0.3500	1.0237
SDR	-0.0041	0.0470	-0.0870	0.9309	-0.0975	0.0893
Int_1	0.0022	0.0038	0.5734	0.5679	-0.0054	0.0098
Age	0.1595	0.1201	1.3281	0.1877	-0.0792	0.3982

IV: Utilization						
Constant	0.5467	1.7393	0.3143	0.7540	-2.9109	4.0043
Knowledge	-0.7118	0.4240	-1.6788	0.0968	-1.5546	0.1311
SDR	0.0313	0.0577	0.5425	0.5889	-0.0833	0.1459
Int_1	0.0132	0.0047	2.8020	0.0063	0.0038	0.0226
Age	-0.3901	0.1474	-2.6472	0.0097	-0.6831	-0.0972

*Note:* Int_1=Interaction effect, IV=Independent variables, p=probability, SDR=Social Desirability Responding, LLCI=Lower limit confidence interval, ULCI=Upper limit confidence interval, TC_1_=One of the teacher college in the treatment group.

**Table 2:** Indicates the moderation of SDR on the relationship between environmental knowledge and environmental attitudes in TC_2_ at the pre-test.

Variables	Coeff.	se	t	p	LLCI	ULCI
IV: Preservation						
Constant	0.2536	1.5699	0.1615	0.8722	-2.8827	3.3900
Knowledge	0.6135	0.3979	1.5417	0.1281	-0.1815	1.4084
SDR	-0.0346	0.0659	-0.5245	0.6017	-0.1662	0.0971
Int_1	0.0043	0.0095	0.4507	0.6537	-0.0148	0.0234
Age	-0.0469	0.2124	-0.2207	0.8261	-0.4711	0.3774

IV: Utilization						
Constant	1.9385	1.9486	0.9948	0.3236	-1.9543	5.8312
Knowledge	-0.7169	0.4939	-1.4516	0.1515	-1.7036	0.2697
SDR	0.0937	0.0818	1.1457	0.2562	-0.0697	0.2570
Int_1	0.0346	0.0118	2.9179	0.0049	0.0109	0.0582
Age	-0.1500	0.2636	-0.5689	0.5714	-0.6766	0.3767

*Note*: Int_1=Interaction effect, IV=Independent variables, p=probability, SDR=Social Desirability Responding, LLCI=Lower limit confidence interval, ULCI=Upper limit confidence interval, TC_2_= One of the teacher college in the comparison group.

## 3 Experimental Design, Materials and Methods

### 3.1 Sampling and Data Collection Procedure

The presented dataset was generated from four teacher colleges (TCs) located in four different regions in Tanzania. For ethical consideration, the four TCs were given pseudonyms: TC_1_, TC_2_, TC_3_, and TC_4_. Initially, TCs that offer science-related diploma programs for secondary education were recognized. Specifically, 10 TCs were identified as potential clusters for data generation. Thereafter, the specific number of required participants for the data collection was decided based on the type of data analysis needed to generate adequate statistical power [9,10]. Given the maximum number of 20 items that were employed in the 2-MEV as the scale with the highest number of items, 200 participants were considered adequate for the proposed analysis techniques. To obtain this number, three TCs would have been adequate given the average number of pre-service biology teachers in each intact class. However, three TCs would not provide a comparable dataset for measuring the impact of differentiated instructional strategies. Therefore, four TCs that were randomly selected using single-stage cluster sampling were reasonably considered for data collection [11], [12], [13].

Furthermore, 357 participants were initially used for data collection. Nonetheless, after a thorough data cleaning, 24 questionnaires were excluded from the dataset, and this resulted in 333 (162 males, 171 females) participants with a complete dataset. The ages of the participants ranged from 17 to 32 (males: M=21.8, SD=2.9; females: M=20.6, SD=2.0; Total: M=21.2, SD=2.6).

### 3.2 The Intervention and Data Analysis Procedure

The primary purpose of the intervention was to assess the effect of an inquiry-based learning (IBL) approach as compared to conventional methods in shaping environmental attitudes and environmental knowledge dimensions in pre-service biology teachers. In so doing, the intervention was carried out using a modified Solomon four-group design. Therefore, two groups (TC_1_ and TC_3_) were exposed to an intervention program and an IBL as an instructional method. The intervention was designed in such a way that pre-service biology teachers were engaged in learning activities through an outdoor learning approach. The study's intervention was built on three frameworks. In the methodological design, the intervention plan and implementation were guided by Experiential Learning Theory (ELT), as proposed by Kolb and Kolb [14]. This theory provides the methodological guidelines in education through which holistic learning takes place between a learner and the surrounding environment. The ELT builds on ideas pioneered by John Dewey and social constructivism with a primary focus on the learner's experience in generating knowledge and solutions to existing challenges (in this context, environmental challenges). ELT is an extension of IBL that takes on a bolder task to engage learners in an interactive way [15]. For measuring EAs, the 2-MEV was used as a framework for assessing the effect of the intervention, as proposed by Bogner and Wiseman [1,16]. Besides, the assessment of environmental knowledge was guided by a Competence Model for Environmental Education adapted from Roczen and colleagues [2] as an extension of their earlier publication [3].

The themes on which the intervention focused were extracted from the syllabus for pre-service biology teachers used in teacher colleges in Tanzania. Specifically, the themes included an exploration of the impact of rapid human population growth on the environment and the effects of environmental pollution on living things [17]. Pre-service biology teachers were engaged in these themes using differentiated facilitation approaches. Those who were in the treatment study groups (TC_1_ and TC_3_) participated in the study through an inquiry-based exploration using outdoor learning activities. Particularly, pre-service biology teachers were interactively engaged with the surrounding environment to explore key issues that included waste management strategies, college-based environmental activities, utility savings, and management of laboratory chemical spills and other hazardous wastes, including disposal of e-wastes (e.g., remains of electronic materials) within the college campuses. Pre-service biology teachers observed and recorded important aspects of outdoor learning. After the outdoor learning activities, they returned to the classrooms for a reflective discussion. On the other hand, the conventional approach was used in two other study groups (TC_2_ and TC_4_), referred to hereafter as the comparison groups. Therefore, the comparison groups continued with their routine instructional approaches without additional intervention strategies, but the same content was administered in both the treatment and the comparison groups. Along with environmental knowledge and EAs, environmental identity was also observed, but its resulting dataset is not part of this article as the related dataset was published in another article cited herein as an article related to the presented dataset [18].

To compare the impacts of the intervention and control for pre- test sensitization [19], one TC from the treatment group (TC_1_) and another from the comparison group (TC_2_) received pre-and post-intervention measurements, while other study groups received post-intervention measurements only (TC_3_ and TC_4_). Thereafter, all groups were compared based on their post-measurement scores. The outcome data on the scores obtained on environmental knowledge and attitudes are presented in the Mendeley data repository through the same link cited earlier in this article (link to the files through: https://data.mendeley.com/datasets/ww6vn5v3dy/1). The files provide mean scores before and after the study intervention in TC_1_ and TC_2_ and post-intervention scores in TC_3_ and TC_4_ because the latter had no pre-test measurement, as stated earlier. The motivation to use differentiated instruction strategies based on previous studies that reported limited exposure of pre-service teachers to practical learning and their mixed beliefs towards learning and generation of new knowledge [20].

Tables 3 and 4 describe the data of the post-intervention measurement by indicating the moderation effect of SDR on the relationships that existed between environmental attitudes and environmental knowledge in the comparison and treatment groups. The data presented in Tables 3 and 4 help to visually indicate the change in the moderation effect of SDR on the relationship between environmental knowledge and environmental attitudes. Nonetheless, the interpretation of the presented data is beyond the scope of the present journal.

**Table 3:** Indicates the moderation of SDR on the relationship between environmental knowledge and environmental attitudes in the comparison groups at the post-test.

Variables	Coeff.	se	t	p	LLCI	ULCI
IV: Preservation						
Constant	-0.0954	0.9495	-0.1005	0.9201	-1.9723	1.7815
Knowledge	0.0800	0.0358	2.2348	0.0270	0.0092	0.1507
SDR	-0.0051	0.0068	-0.7523	0.4531	-0.0185	0.0083
Int_1	0.0004	0.0002	2.5121	0.0131	0.0001	0.0008
Age	0.0067	0.0173	0.3861	0.7000	-0.0275	0.0409

IV: Utilization						
Constant	-0.0073	1.1563	-0.0063	0.9950	-2.2931	2.2786
Knowledge	0.0154	0.0436	0.3532	0.7245	-0.0708	0.1016
SDR	-0.0146	0.0083	-1.7729	0.0784	-0.0310	0.0017
Int_1	0.0000	0.0002	0.1882	0.8510	-0.0004	0.0005
Age	-0.0008	0.0211	-0.0358	0.9715	-0.0424	0.0409

*Note*: Int_1=Interaction effect, IV=Independent variables, p=probability, SDR=Social Desirability Responding, LLCI=Lower limit confidence interval, ULCI=Upper limit confidence interval.

**Table 4:** Indicates the moderation of SDR on the relationship between environmental knowledge and EA in the treatment study groups at the post-test.

	Coeff.	se	t	p	LLCI	ULCI
IV: Preservation						
Constant	0.0494	0.8079	0.0611	0.9513	-1.5448	1.6435
Knowledge	0.1177	0.0307	3.8355	0.0002	0.0572	0.1783
SDR	-0.0064	0.0057	-1.1101	0.2684	-0.0177	0.0049
Int_1	0.0002	0.0001	1.6018	0.1109	-0.0001	0.0005
Age	0.0020	0.0122	0.1605	0.8727	-0.0221	0.0260

IV: Utilization						
Constant	-0.0164	0.9390	-0.0175	0.9861	-1.8692	1.8364
Knowledge	-0.1562	0.0357	-4.3772	0.0000	-0.2266	-0.0858
SDR	0.0047	0.0067	0.7030	0.4830	-0.0085	0.0178
Int_1	-0.0001	0.0002	-0.5417	0.5887	-0.0004	0.0002
Age	0.0002	0.0142	0.0139	0.9889	-0.0278	0.0282

*Note*: Int_1=Interaction effect, IV=Independent variables, p=probability, SDR=Social Desirability Responding, LLCI=Lower limit confidence interval, ULCI=Upper limit confidence interval.

**Fig. 3:**
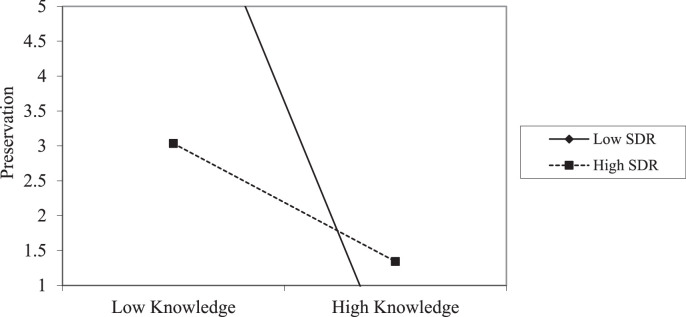
Indicates the interaction effect of SDR on the relationship between environmental knowledge and Preservation in the comparison groups at the post-test. To effectively use this data, the information processing approach can be useful**.**

It is worth noting that in all the assessments of the relationships between variables, age was controlled as a covariate when the assessment of the interaction effect of SDR on the relationships of the variables was conducted, given its potential impacts on self-reported responses when measuring environmental constructs, as emphasized by Wiseman and Bogner [16]. In addition, Fig. 3 presents a graphical indication of the moderation effect of SDR on the relationships between EAs and environmental knowledge at the post-tests in comparison groups. Fig. 3 helps to provide a visual indication of the level of interaction effect that SDR presents when environmental knowledge interacts with EAs, particularly the Preservation of nature.

## Ethics Statements

This study was conducted according to the guidelines of the Declaration of Helsinki, and approved by the Institutional Review Board of the College of Education of the University of Rwanda (Protocol # 01/P-CE/745/ENg/2019 approved September 23, 2019).

Before engaging participants, informed consent was obtained from the teacher colleges' management office, which holds responsibility for the guardianship of pre-service teachers during their stay at the colleges. Then, participants filled out consent forms after getting permission from their colleges' leaders for their participation in generating the presented dataset.

## CRediT authorship contribution statement

**Josephat Paul Nkaizirwa:** Conceptualization, Investigation, Methodology, Software, Data curation, Writing – original draft, Validation. **Catherine Musalagani Aurah:** Writing – review & editing, Supervision, Validation. **Florien Nsanganwimana:** Writing – review & editing, Supervision, Validation.

## Data Availability

Data for assessing EA and knowledge in selected teacher colleges in Tanzania (Reference data) (Mendeley Data).
