# Corrigendum to “Data collected to assess the effect of inquiry-based learning on environmental knowledge and attitudes among pre-service biology teachers in Tanzania” [Data in Brief, 49 (August, 2023), 1–10/ 109429]

**DOI:** 10.1016/j.dib.2023.109495

**Published:** 2023-08-12

**Authors:** Josephat Paul Nkaizirwa, Catherine Musalagani Aurah, Florien Nsanganwimana

**Affiliations:** a African Centre of Excellence for Innovative Teaching and Learning Mathematics and Science (ACEITLMS), University of Rwanda, College of Education (URCE), P.O. Box 55, Kayonza, Rwamagana, Rwanda; b Department of Educational Psychology and Curriculum Studies, College of Education, The University of Dodoma, P.O. Box 523, Dodoma, Tanzania; c Department of Science and Mathematics Education, School of Education, Masinde Muliro University of Science and Technology, P.O. Box 190-50100, Kakamega, Kenya

The authors regret to make some minor changes in the published articles particularly in the Tables. Author would like to correct abbreviations and annotations Table 1, Table 2, Table 3, Table 4. The abbreviations IV in Tables 1 and 2 on page 5 and in Tables 3 and 4 on page 8. IV should be replaced with DV, whereas independent variable as an annotation for IV should be replaced with dependent variable as an annotation for DV. The corrected Tables as presented after corrections as follows;

**Table 1:** Indicates the moderation of SDR on the relationship between environmental knowledge and environmental attitudes in TC_1_ at the pre-test.

Variables	Coeff.	se	t	p	LLCI	ULCI
DV: Preservation						
Constant	.4524	1.4174	.3192	.7504	-2.3653	3.2701
Knowledge	.3369	.3455	.9750	.3323	-.3500	1.0237
SDR	-.0041	.0470	-.0870	.9309	-.0975	.0893
Int_1	.0022	.0038	.5734	.5679	-.0054	.0098
Age	.1595	.1201	1.3281	.1877	-.0792	.3982

DV: Utilization						
Constant	.5467	1.7393	.3143	.7540	-2.9109	4.0043
Knowledge	-.7118	.4240	-1.6788	.0968	-1.5546	.1311
SDR	.0313	.0577	.5425	.5889	-.0833	.1459
Int_1	.0132	.0047	2.8020	.0063	.0038	.0226
Age	-.3901	.1474	-2.6472	.0097	-.6831	-.0972

*Note*: Int_1 = Interaction effect, DV = Dependent variables, *p* = probability, SDR = Social Desirability Responding, LLCI = Lower limit confidence interval, ULCI = Upper limit confidence interval, TC_1_ = One of the teacher college in the treatment group

**Table 2:** Indicates the moderation of SDR on the relationship between environmental knowledge and environmental attitudes in TC_2_ at the pre-test.

Variables	Coeff.	se	t	p	LLCI	ULCI
DV: Preservation						
Constant	.2536	1.5699	.1615	.8722	-2.8827	3.3900
Knowledge	.6135	.3979	1.5417	.1281	-.1815	1.4084
SDR	-.0346	.0659	-.5245	.6017	-.1662	.0971
Int_1	.0043	.0095	.4507	.6537	-.0148	.0234
Age	-.0469	.2124	-.2207	.8261	-.4711	.3774

DV: Utilization						
Constant	1.9385	1.9486	.9948	.3236	-1.9543	5.8312
Knowledge	-.7169	.4939	-1.4516	.1515	-1.7036	.2697
SDR	.0937	.0818	1.1457	.2562	-.0697	.2570
Int_1	.0346	.0118	2.9179	.0049	.0109	.0582
Age	-.1500	.2636	-.5689	.5714	-.6766	.3767

*Note*: Int_1 = Interaction effect, DV = Dependent variables, *p* = probability, SDR = Social Desirability Responding, LLCI = Lower limit confidence interval, ULCI = Upper limit confidence interval, TC_2_ = One of the teacher college in the comparison group

**Table 3:** Indicates the moderation of SDR on the relationship between environmental knowledge and environmental attitudes in the comparison groups at the post-test.

Variables	Coeff.	se	t	p	LLCI	ULCI
DV: Preservation						
Constant	-.0954	.9495	-.1005	.9201	-1.9723	1.7815
Knowledge	.0800	.0358	2.2348	.0270	.0092	.1507
SDR	-.0051	.0068	-.7523	.4531	-.0185	.0083
Int_1	.0004	.0002	2.5121	.0131	.0001	.0008
Age	.0067	.0173	.3861	.7000	-.0275	.0409

DV: Utilization						
Constant	-.0073	1.1563	-.0063	.9950	-2.2931	2.2786
Knowledge	.0154	.0436	.3532	.7245	-.0708	.1016
SDR	-.0146	.0083	-1.7729	.0784	-.0310	.0017
Int_1	.0000	.0002	.1882	.8510	-.0004	.0005
Age	-.0008	.0211	-.0358	.9715	-.0424	.0409

*Note*: Int_1 = Interaction effect, DV = Dependent variables, p=probability, SDR = Social Desirability Responding, LLCI = Lower limit confidence interval, ULCI = Upper limit confidence interval.

**Table 4:** Indicates the moderation of SDR on the relationship between environmental knowledge and EA in the treatment study groups at the post-test.

	Coeff.	se	t	p	LLCI	ULCI
DV: Preservation						
Constant	.0494	.8079	.0611	.9513	-1.5448	1.6435
Knowledge	.1177	.0307	3.8355	.0002	.0572	.1783
SDR	-.0064	.0057	-1.1101	.2684	-.0177	.0049
Int_1	.0002	.0001	1.6018	.1109	-.0001	.0005
Age	.0020	.0122	.1605	.8727	-.0221	.0260

DV: Utilization						
Constant	-.0164	.9390	-.0175	.9861	-1.8692	1.8364
Knowledge	-.1562	.0357	-4.3772	.0000	-.2266	-.0858
SDR	.0047	.0067	.7030	.4830	-.0085	.0178
Int_1	-.0001	.0002	-.5417	.5887	-.0004	.0002
Age	.0002	.0142	.0139	.9889	-.0278	.0282

*Note*: Int_1 = Interaction effect, DV = Dependent variables, *p* = probability, SDR = Social Desirability Responding, LLCI = Lower limit confidence interval, ULCI = Upper limit confidence interval.

The authors would like to apologise for any inconvenience caused.

